# Lower objectively and subjectively assessed numeracy are both associated with poorer self-rated health

**DOI:** 10.1186/s13104-021-05737-y

**Published:** 2021-08-21

**Authors:** Natalie C. Benda, Zihan Yang, Haojia Li, Tianran Zhang, Jessica S. Ancker

**Affiliations:** 1 Department of Population Health Sciences, Weill Cornell Medicine, 425 E 61 stStreet, Suite 301, New York, NY 10065 USA; 2 Department of Biomedical Informatics, Vanderbilt University Medical Center, 2525 West End Ave., Rm, Nashville, TN 14122 USA

**Keywords:** Numeracy, Health communication, Health status, Social determinants of health

## Abstract

**Objective:**

To compare an objective with a subjective numeracy assessment for association with self-reported health status, where numeracy refers to “the degree to which individuals have the capacity to access, process, interpret, communicate, and act on numerical, quantitative, graphical, biostatistical, and probabilistic health information needed to make effective health decisions”

**Results:**

We completed a secondary analysis of two population-based surveys, the Empire State Poll (n = 763) and the Program for the International Assessment of Adult Competencies (PIAAC; n = 2609). The first survey assessed numeracy with a 3-item subjective instrument. The second assessed numeracy with more than 20 math problems. Both used the same measure for self-reported health status. Lower numeracy, whether subjectively or objectively assessed, was associated with worse self-reported health, even after controlling for education and other sociodemographic confounders. The odds ratios for the association were very similar (0.91 and 0.90 respectively). A lengthy objective numeracy assessment and a brief self-report assessment had similar associations with health status. A brief self-report measure of numeracy has similar properties to a lengthy objective assessment and is likely to be more feasible to use to screen patients in practice.

**Supplementary Information:**

The online version contains supplementary material available at 10.1186/s13104-021-05737-y.

## Introduction

The primary objective of this work was to assess the association between self-rated health status and numeracy using two different approaches for assessing numeracy—a short subjective scale and a longer objective numeracy instrument.

Many individuals throughout the world have low health numeracy, defined as “the degree to which individuals have the capacity to access, process, interpret, communicate, and act on numerical, quantitative, graphical, biostatistical, and probabilistic health information needed to make effective health decisions” [1]. People with lower numeracy have demonstrably poorer health knowledge, self-care practices, and health outcomes [2–4].

Identifying patients with low health numeracy could allow communicators to adapt their information to the needs of the patient by simplifying or providing additional explanations for quantitative concepts [5–7]. Instruments for measuring numeracy and health numeracy range from long, objective examinations to short subjective scales [8–16]. Although short subjective assessments are likely to be more acceptable to patients, are more feasible to apply in clinical practice, and are correlated with objective measures [8, 9], it is necessary to show that they are in fact associated with health status. Previous studies have compared objective (e.g. Lipkus et al., 2001) and subjective measures of numeracy (e.g. Fagerlin et al.’s short numeracy scale known as the SNS-8), though they only included single items from the objective assessments, other than the entire, psychometrically evaluated battery [17–19]. Dolan and colleagues also assessed the relationship between the SNS-8 and various other objective measures of health numeracy, but did not correlate these with health-related constructs such as self-rated health [20].

In this study, we present results of two numeracy assessments of two population-based samples and compare the relationship between numeracy, self-rated health, and sociodemographic variables. We hypothesized that numeracy would be positively associated with self-rated health status using both a short, subjective survey and the longer objective survey.

## Main text

### Methods

One data set is from the Empire State Poll (ESP), a study conducted annually to understand community, economic, and social science issues among New York State residents, with questions submitted by academic researchers. The Survey Research Institute (SRI) of Cornell University conducted all surveys. Our team submitted a validated, three-question, subjective numeracy assessment, known as the SNS-3, and a question on self-rated health status (see Table 1) [16]. The SNS-3 was derived from the previously validated Subjective Numeracy Scale 8 (SNS-8) [8]. The SNS-8 was validated by demonstrating that higher scores on the SNS-8 predicted better performance on multiple, objective quantitative problems [8] The validation study of the SNS-3 demonstrated that it had high internal validity (median Cronbach’s α = 0.78), a high correlation with the SNS-8 (median ρ = 0.91), and statistically significant correlations with multiple other objective measures of quantitative skills and literacy [16].

**Table 1:** Numeracy assessment questions

SNS-3 questions (all)
1. How good are you at working with fractions? 2. How good are you at figuring out how much a shirt will cost if it is 25% off? 3. How often do you find numerical information to be useful?
PIAAC numeracy assessment questions (examples)
1. If the temperature shown [thermometer pictured showing 26º Celsius/78º Fahrenheit], decreases by 30º Celsius, what would the temperature be in degrees Celsius? 2. [Given a line graph showing the number of births per decade]. During which period was there a decline in births? Click all that apply

The second data set is the Program for the International Assessment of Adult Competencies (PIAAC), an international assessment conducted by the intergovernmental Organization for Economic Co-Operation and Development. The PIAAC is conducted to assess basic competencies in a number of skills, including numeracy (our focus), literacy, and problem solving.

ESP participants included New York State residents aged 18 and over who spoke English or Spanish proficiently. Responses were balanced between upstate and downstate residents (New York City area, or “downstate,” and the remainder of the state, or “upstate”). Cornell SRI recruited 800 participants via a random-digit-dial telephone survey between February and April in 2019. The ESP is approved by the Cornell University Institutional Review Board. Data are included in Additional files 1 and 2.

The PIAAC survey included United States residents aged 16–74 years living in households or group quarters, which does not account for persons living in shelters, the incarcerated, military personnel who live in barracks or bases, or persons who live in institutionalized group quarters, such as hospitals or nursing homes. Those aged under 18 years were excluded from our analysis. The PIAAC U.S. 2017 involves a stratified random sample intended to allow for nation and state-level estimates of basic skills. Data were collected between March and November of 2017. Participants were first administered a screener via phone to determine eligibility. Eligible consenting participants completed an in-person background questionnaire then completed competency assessments using a computer or paper-and-pencil (reserved for those who would not or could not use a computer). PIAAC data are publicly available at http://www.oecd.org.

Our primary dependent variable was self-rated health, measured with the same SF-36 question in ESP and PIAAC: “In general, would you say your health is excellent, very good, good, fair, or poor?”[21] Participants could also respond “Do not know” or decline to answer. Our primary independent variable was numeracy. In the ESP, numeracy was assessed with the SNS-3 questions (Table 1), answered on a six-point Likert scale. Likert scale responses are converted to numbers and summed to produce a single score [16]. SNS-3 scores can range from 3 to 18, with higher scores indicating higher subjective numeracy.

The PIAAC measures numeracy using 24 (paper-based) to 52 (computer-based) items covering broad contexts, cognitive processes, and mathematical content (examples in Table 1). Scores are computed as plausible values, a method that has been demonstrated to provide more accurate estimates of group proficiency than single point estimation methods [22, 23]. The PIAAC numeracy score (i.e., average of the 10 plausible values for the sample) can range from 0 to 500, with higher scores indicating higher objective numeracy.

Our analysis also included survey responses to demographic questions involving gender, age, education, income, race, and ethnicity from both surveys [24, 25].

All analyses were conducted using R software version 4.0 (R Core Team, 2018). We first assessed differences in sample demographics between the ESP and PIAAC data sets using Fisher’s exact tests. For both samples, we then created logistic models of the relationship between self-rated health and numeracy, with sociodemographic covariates. We dichotomized self-rated health into not good (poor, fair) and good (good, very good, and excellent) after a preliminary analysis suggested this split improved the AUC and predictive power of the model. For ESP data, weights were added for education, income, race, and ethnicity variables based on a comparison between demographics of the sample and the state using Fisher’s exact tests. The PIAAC was already stratified by gender, age, education, income, race, and ethnicity to reflect the demographics of the United States, so weighting was not necessary [26].

Because the ESP score could range from 3 to 18 (i.e., it had 15 score increments), we also binned the PIAAC numeracy score into 15 increments by creating score categories of 0 to 32, 33 to 65, 66 to 98, etc., up to 500. This improved comparability of the analyses by ensuring that a single increment in numeracy score represented 1/15 of the total possible range in both cases.

Variable selection was conducted on all data based on the model with the lowest Akaike information criterion using stepwise selection in both forward and backward directions using the glm functions from the R stats packages. We randomly split the data into training and test datasets in ratio of 7:3, used the selected variables to build the model on the training data, and applied it on the test data to obtain the AUC and the prediction error (misclassification) rate.

### Results

For the ESP and PIAAC data, 763 and 2609 participants had complete demographic and numeracy data, respectively (Table 2). There were significant differences in the demographic distribution across all categories analyzed, except for race. PIAAC participants with incomplete data for problem solving competencies were also excluded (analysis not presented here). Low numeracy was prevalent in both samples, with 24.8% of ESP participants in the lowest category of SNS-3 scores, and 22.8% of the PIAAC participants in the lowest two categories of numeracy (Table 2).

**Table 2:** Demographic characteristics of each sample

	ESP		PIAAC	
	(N = 763)		(N = 2609)	
Gender*				
Male	389 (51.0%)		1195 (45.8%)	
Female	374 (49.0%)		1414 (54.2%)	
Age**				
18–24	90 (11.8%)		368 (14.1%)	
25–44	239 (31.3%)		1060 (40.6%)	
45–64	274 (35.9%)		970 (37.2%)	
65 +	160 (21.0%)		211 (8.1%)	
Education**				
High school or less	173 (22.7%)		1106 (42.4%)	
Some college or associate's degree	216 (28.3%)		508 (19.5%)	
Bachelor's degree or higher	374 (49.0%)		995 (38.1%)	
Income***				
< $50 k	252 (33.0%)		984 (37.7%)	
$50 k–$100 k	259 (33.9%)		889 (34.1%)	
$100 k–$150 k	109 (14.3%)		411 (15.8%)	
$150 k +	143 (18.7%)		325 (12.5%)	
Race				
White	522 (68.4%)		1800 (69.0%)	
Black	98 (12.8%)		319 (12.2%)	
Other	143 (18.7%)		490 (18.8%)	
Ethnicity***				
Hispanic	118 (15.5%)		281 (10.8%)	
Non-Hispanic	645 (84.5%)		2328 (89.2%)	
Self-rated health***				
Poor	26 (3.4%)		62 (2.4%)	
Fair	126 (16.5%)		318 (12.2%)	
Good	275 (36.0%)		800 (30.7%)	
Very good	234 (30.7%)		932 (35.7%)	
Excellent	102 (13.4%)		497 (19.0%)	
Numeracy scores				
SNS-3 score category	3–12	188 (24.6%)	–	–
	13–15	249 (32.6%)	–	–
	16–18	326 (42.8%)	–	–
PIAAC score category^†^	–	–	0–175	126 (4.8%)
	–	–	176–225	448 (17.1%)
	–	–	226–275	909 (34.8%)
	–	–	276–325	858 (32.9%)
			326–375	252 (9.7%)
			376–500	16 (0.6%)

*Signifies p < 0.05; **Signifies p < 0.01; ***Signifies p < 0.001

^†^Based on levels defined by PIAAC [27]

### Multivariate analyses

Table 3 presents the results of the final logistic regression models, including their respective power and AUC. As shown, in both ESP and PIAAC, self-rated health was significantly associated with numeracy, even in models that controlled for age, education, and income.

**Table 3:** Multivariate logistic regression of self-rated health status as function of numeracy and sociodemographic variables

Variable	ESP (power = 0.81, AUC = 0.72)		PIAAC (power = 0.60, AUC = 0.69)	
	OR	95% CI	OR	95% CI
Numeracy	0.91*	(0.86, 0.97)	0.90*	(0.827, 0.983)
Gender (female)	0.66*	(0.45, 0.98)	–	–
Age				
18–24	Ref		Ref	
25–44	3.60***	(1.83, 7.51)	1.3	(0.89, 1.91)
45–64	4.14***	(2.10, 8.64)	2.35***	(1.63, 3.46)
65 +	5.27***	(2.60,11.26)	2.52***	(1.54, 4.12)
Education				
High school or less	Ref			
Some college or associate's degree	0.41***	(0.25, 0.66)	0.69*	(0.52, 0.94)
Bachelor's degree or higher	0.36***	(0.22, 0.58)	0.52***	(0.38, 0.72)
Income				
< $50 k	Ref			
$50 k– < $100 k	0.35***	(0.21, 0.58)	0.50***	(0.38, 0.66)
$100 k- < $150 k	0.53*	(0.29, 0.94)	0.32***	(0.20, 0.48)
$150 k +	0.42**	(0.21, 0.78)	0.34***	(0.21, 0.54)
Race				
White	Ref			
Black	1.47	(0.91, 2.36)	–	–
Other	0.72	(0.39, 1.27)	–	–
Ethnicity				
Hispanic	Ref			
Non-Hispanic	0.42***	(0.25, 0.68)	–	–

*Signifies p < 0.05; **Signifies p < 0.01; ***Signifies p < 0.001

As hypothesized, lower objective/subjective numeracy was associated with lower self-rated health in both models. The odds ratios show that a single point decrease in the SNS-3 score was associated with 9% lower odds of having good health; similarly, a single category decrease in PIAAC numeracy score was associated with a 10% lower odds of good health. Other variables associated with lower self-rated health in both models included: higher age, lower education, and lower income. In the ESP model only, male gender and Hispanic ethnicity were associated with lower self-rated health.

### Discussion

Numeracy assessed subjectively with a brief 3-item instrument is associated with self-rated health, even after controlling for education and other sociodemographic variables. The association is similar to the association found when assessing numeracy with a complex objective assessment, which would be burdensome to administer in clinical practice. With the subjective and the objective assessments, a single increment in numeracy score was associated with an odds ratio of approximately 0.90, indicating about 10% reduced odds of good self-reported health. The similarity in results suggest that it is not always necessary to evaluate numeracy with a burdensome objective scale, but that a short subjective scale could instead be used in clinical practice. In addition, the brief subjective questions are simple to score and interpret.

These two analyses also replicated previous findings confirming that younger age, higher income, and higher education were all associated with both higher numeracy and better self-rated health. All of these relationships were in the direction that would be expected from previous research [28–30]. However, the association between low numeracy and poor health is not fully attributable to these other variables as it remained significant even after controlling for the demographic variables. There were significant differences in the demographic makeup of the two samples. While observation of statistically significant differences may be expected in samples as large those in the PIAAC data, it may be beneficial to have more balanced demographic samples, perhaps representing the same target population (instead of New York state vs. the entire United States), in future studies.

It could be helpful to begin including questions related to literacy/numeracy in initial visits to help health professionals better tailor their communication. Once patients with low numeracy are identified, a growing body of research demonstrates how rewriting patient information can help [31]. For example, Davis et al. demonstrated that rewording medication instructions by phrasing instructions in terms of time of day rather than pills per day disproportionately improves comprehension among low numeracy patients [32], and Yin and colleagues have shown that low-literacy patients benefit from pictogram-enhanced medication instructions (33).

## Limitations

The study has several limitations. The ESP and PIAAC were conducted for different purposes in different populations. The ESP sample was also found to be significantly different in demographic distribution from the NYS population, which was handled using survey weights.

## Supplementary Information

**Additional file 1.** Raw Empire State poll data.

**Additional file 2.** Codebook for raw data file, Empires State Poll data.

## Data Availability

All data generated or analysed during this study are included in this published article [and its additional files]. Empire State Poll data is included as additional material, and the PIAAC data is publicly available at http://www.oecd.org.

## Abbreviations

SNS: Subjective Numeracy Scale
ESP: Empire State Poll
SRI: Survey Research Institute
PIAAC: Program for the International Assessment of Adult Competencies
AUC: Area Under the Curve
OR: Odds ratio
CI: Confidence interval
