# Supplementary material for: Lower objectively and subjectively assessed numeracy are both associated with poorer self-rated health
Source: BMC Res Notes. 2021 Aug 21;14:321. doi: 10.1186/s13104-021-05737-y (PMC8379725; doi:10.1186/s13104-021-05737-y)
Supplement: Supplementary file 2 — Additional file 2. Codebook for raw data file, Empires State Poll data. [file 13104_2021_5737_MOESM2_ESM.doc]

CODEBOOK

Empire State Poll 2019

800 Cases

**4/30/2019


Prepared by:

Survey Research Institute (SRI) at Cornell
391 Pine Tree Road, Room 118
Ithaca, NY 14850
Tel (607) 255-3786
Fax (607) 255-7118
www.sri.cornell.edu**

**INTRODUCTION**

The 2019 Empire State Poll (ESP 2019) is the seventeenth annual general survey of New York State residents age 18 and over. It is an omnibus study combining an annual core of community, economic, and social science modules and questions submitted by academic researchers. The ESP 2019 was conducted by the Survey Research Institute at Cornell University (SRI).

The survey sample consists of a dual-frame RDD sample covering both cellular and landline exchanges for New York State. The phone numbers are purchased from Marketing Systems Group. Once a household is sampled, every adult has an equal chance to be included in the poll. With 800 respondents (400 Upstate and 400 Downstate), in no more than one time in twenty should chance variations in the sample cause the overall ESP 2019 results to vary by more than 3.5 percentage points from the results that would be obtained if all New York state residents were interviewed. The sampling frame is split between Upstate and Downstate residents, allowing comparisons between the whole state and these geographic regions with a one in twenty chance of sampling error greater than 4.9 percentage points.

The random sampling frame used within the ESP 2019 allows the poll results to be generalized to the entire state. Such generalizations may be made by using the provided weight variable, though all results reported here are based on un-weighted data.

Telephone data collection for the full study began on February 18, 2019 and ended on April 23, 2019. Interviews were conducted in English and Spanish using a Computer Assisted Telephone Interviewing (CATI) software system. The average interview length was 28 minutes.

N = 800

filename = alldata.dat

file type = fixed-format ASCII

logical record length = 409

**DEFINITIONS**

INPUT LOCATION = Location of variable within data set. In card-image format, this would be card/column location.

VALUE = Numeric value given to each discrete response category. May also reflect the quantitative value of a continuous variable.

NUMBER (N) = Frequency of response.

PERCENT (PCT) = Percentage of response.

MISSING DATA (MD) = Code value given to any question which was unanswered or refused by the respondent.

VALUE = blank = The variable field is blank in the data set because the question does not apply. Typically these are questions embedded within a skip pattern.

Contents

**phone**: Phone (provided by MSG) [1](#__RefHeading___Toc7522900)

**WEIGHT**: Geographic weight (assigned by SRI) [1](#__RefHeading___Toc7522901)

**CITY**: City (provided by MSG) [1](#__RefHeading___Toc7522902)

**STATE**: State (provided by MSG) [1](#__RefHeading___Toc7522903)

**ZIP**: ZIP code (provided by MSG) [2](#__RefHeading___Toc7522904)

**FIPS**: FIPS (provided by MSG) [2](#__RefHeading___Toc7522905)

**MSC**: Metropolitan MSC (provided by MSG) [2](#__RefHeading___Toc7522906)

**CENREG**: Census Region (provided by MSG) [3](#__RefHeading___Toc7522907)

**CENDIV**: Census Division (provided by MSG) [3](#__RefHeading___Toc7522908)

**CBSA**: CBSA CODE 2016 (provided by MSG) [4](#__RefHeading___Toc7522909)

**CBSADIV**: CBSA DIV CODE 2016 (provided by MSG) [5](#__RefHeading___Toc7522910)

**CENTRACT**: Census Tract - Primary (provided by MSG) [5](#__RefHeading___Toc7522911)

**nysregion**: NYS region (Upstate/Downstate) [6](#__RefHeading___Toc7522912)

**county**: NYS county (reported by R) [6](#__RefHeading___Toc7522913)

**updown**: NYS region (reported by R) [7](#__RefHeading___Toc7522914)

**nysimp**: Most important NYS issue [8](#__RefHeading___Toc7522915)

**finpast**: Past year - Personal finances [9](#__RefHeading___Toc7522916)

**finfut**: Next year - Personal finances [9](#__RefHeading___Toc7522917)

**econpast**: Past year - NYS economy [10](#__RefHeading___Toc7522918)

**econfut**: Next year - NYS economy [10](#__RefHeading___Toc7522919)

**JAq1**: Fractions - Rate competence [11](#__RefHeading___Toc7522920)

**JAq2**: Percentages - Rate competence [11](#__RefHeading___Toc7522921)

**JAq3**: Numerical information - Rate usefulness [12](#__RefHeading___Toc7522922)

**JAq4**: Rate health [12](#__RefHeading___Toc7522923)

**SRIq1**: Societal pressure to attend college [13](#__RefHeading___Toc7522924)

**SRIq2**: Reason for change in societal pressure to attend college [13](#__RefHeading___Toc7522925)

**SRIq3**: Parents education [14](#__RefHeading___Toc7522926)

**trst@corp**: Large corporations - Trustworthiness [15](#__RefHeading___Toc7522927)

**trst@bsns**: Local business - Trustworthiness [16](#__RefHeading___Toc7522928)

**trst@lgov**: Local gov - Trustworthiness [17](#__RefHeading___Toc7522929)

**trst@sgov**: State gov - Trustworthiness [18](#__RefHeading___Toc7522930)

**trst@union**: Labor unions - Trustworthiness [19](#__RefHeading___Toc7522931)

**trst@media**: News media - Trustworthiness [20](#__RefHeading___Toc7522932)

**trst@ppl**: Other people - Trustworthiness [21](#__RefHeading___Toc7522933)

**trst@intrnt**: Internet - Trustworthiness [22](#__RefHeading___Toc7522934)

**employ**: Employment status [23](#__RefHeading___Toc7522935)

**jbtype**: Main job type [23](#__RefHeading___Toc7522936)

**hrswrk**: Hours worked last week [24](#__RefHeading___Toc7522937)

**slfempl**: Self-employed [24](#__RefHeading___Toc7522938)

**lkwork**: Looking for new work [25](#__RefHeading___Toc7522939)

**bornus**: Born in US or to an American [25](#__RefHeading___Toc7522940)

**yob**: Year of birth [26](#__RefHeading___Toc7522941)

**age**: Age (computed from yob) [26](#__RefHeading___Toc7522942)

**Zipcode**: ZIP code [26](#__RefHeading___Toc7522943)

**married**: Marital status [27](#__RefHeading___Toc7522944)

**ideo**: Social ideology [27](#__RefHeading___Toc7522945)

**party**: Political party [28](#__RefHeading___Toc7522946)

**educ**: Education level [28](#__RefHeading___Toc7522947)

**SRIq4**: Reason NOT to attend college [29](#__RefHeading___Toc7522948)

**SRIq5**: Reason to attend college [29](#__RefHeading___Toc7522949)

**hisp**: Hispanic or Latino [30](#__RefHeading___Toc7522950)

**race@white**: White/Caucasian - Race [30](#__RefHeading___Toc7522951)

**race@black**: Black/African American - Race [31](#__RefHeading___Toc7522952)

**race@natam**: American Indian/Aleut/Eskimo - Race [31](#__RefHeading___Toc7522953)

**race@asian**: Asian/Pacific Islander - Race [32](#__RefHeading___Toc7522954)

**race@other**: Other - Race [32](#__RefHeading___Toc7522955)

**relig**: Religious affiliation [33](#__RefHeading___Toc7522956)

**church**: How often attend religious services [33](#__RefHeading___Toc7522957)

**hhsize@65**: # Adults 65+ in household [34](#__RefHeading___Toc7522958)

**hhsize@1864**: # Adults 18-64 in household [34](#__RefHeading___Toc7522959)

**hhsize@chld**: # Children in household [35](#__RefHeading___Toc7522960)

**hhince**: Exact household income [35](#__RefHeading___Toc7522961)

**hhinc50k**: Over/Under $50k - Household income [36](#__RefHeading___Toc7522962)

**hhincu**: Range under $50k - Household income [36](#__RefHeading___Toc7522963)

**hhinco**: Range over $50k - Household income [37](#__RefHeading___Toc7522964)

**hhinc**: Household income - Final coded value [38](#__RefHeading___Toc7522965)

**gender**: Gender [39](#__RefHeading___Toc7522966)

**datecomp@month**: Month - Date of completion [39](#__RefHeading___Toc7522967)

**datecomp@day**: Day - Date of completion [40](#__RefHeading___Toc7522968)

**datecomp@year**: Year - Date of completion [41](#__RefHeading___Toc7522969)

**datecomp@time**: Time started [41](#__RefHeading___Toc7522970)

**survlang**: Survey language [42](#__RefHeading___Toc7522971)

**phone**: Phone (provided by MSG)

Phone (provided by MSG)

800 cases

Data type: character

Record/columns: 1/6-15

**WEIGHT**: Geographic weight (assigned by SRI)

Geographic weight (assigned by SRI)

% N VALUE LABEL

50.0 400 0.72

50.0 400 1.28

----- ---

100.0 800 cases

Data type: character

Record/columns: 1/16-24

**CITY**: City (provided by MSG)

City (provided by MSG)

800 cases

Data type: character

Record/columns: 1/25-44

**STATE**: State (provided by MSG)

State (provided by MSG)

% N VALUE LABEL

100.0 800 NY

----- ---

100.0 800 cases

Data type: character

Record/columns: 1/45-46

**ZIP**: ZIP code (provided by MSG)

ZIP code (provided by MSG)

800 cases

Data type: character

Record/columns: 1/47-51

**FIPS**: FIPS (provided by MSG)

The numerical code assigned to a county.

800 cases

Data type: character

Record/columns: 1/52-56

**MSC**: Metropolitan MSC (provided by MSG)

The MET Status code is a one-digit code, assigned by MSG. It takes the exchange

and describes the area in relationship to the MSA.

1 In the Center City of an MSA

2 Outside the Center City of an MSA (but inside county containing the Center City)

3 Inside a Suburban County of the MSA

4 In an MSA that has NO Center City

5 Not in an MSA

% N VALUE LABEL

11.6 93 1 In the Center City of an MSA

2.1 17 2 Outside the Center City of an MSA...

3.9 31 3 Inside a Suburban County of the MSA

1.9 15 5 Not in an MSA

80.5 644

----- ---

100.0 800 cases

Data type: character

Record/column: 1/57

**CENREG**: Census Region (provided by MSG)

Census Region is a geographic area consisting of several States defined

by the U.S. Department of Commerce, Bureau of the Census. The States

are grouped into four regions.

% N VALUE LABEL

100.0 800 1 Northeast

----- ---

100.0 800 cases

Data type: character

Record/column: 1/58

**CENDIV**: Census Division (provided by MSG)

Census Division is a geographic area consisting of several States defined

by the U.S. Department of Commerce, Bureau of the Census. The States are

grouped into four regions and then subdivided into 9 divisions.

% N VALUE LABEL

100.0 800 2 Middle Atlantic

----- ---

100.0 800 cases

Data type: character

Record/column: 1/59

**CBSA**: CBSA CODE 2016 (provided by MSG)

Based on 2016 codes. Consist of the county or counties or equivalent entities

associated with at least one core (urbanized area or urban cluster) of at

least 10,000 population, plus adjacent counties having a high degree of

social and economic integration with the core as measured through commuting

ties with the counties associated with the core

% N VALUE LABEL

6.4 51 10580

1.0 8 12180

0.3 2 12860

2.8 22 13780

7.4 59 15380

0.5 4 18500

0.6 5 18660

1.3 10 21300

1.5 12 24020

0.1 1 24100

0.1 1 26460

1.5 12 27060

1.4 11 27460

0.8 6 28740

0.1 1 31660

55.0 440 35620

0.8 6 36300

0.3 2 36460

1.0 8 38460

7.6 61 40380

0.1 1 42900

4.8 38 45060

2.3 18 46540

0.8 6 48060

1.9 15

----- ---

100.0 800 cases

Data type: character

Record/columns: 1/60-64

**CBSADIV**: CBSA DIV CODE 2016 (provided by MSG)

Based on 2016 codes, are smaller groupings of counties or equivalent entities

defined within a metropolitan statistical area containing a single core with a

population of at least 2.5 million. Not all metropolitan statistical areas with

urbanized areas of this size will contain metropolitan divisions. A metropolitan

division consists of one or more main/secondary counties that represent an

employment center or centers, plus adjacent counties associated with the

main/secondary county or counties through commuting ties. Because metropolitan

divisions represent subdivisions of larger metropolitan statistical areas,

it is not appropriate to rank or compare metropolitan divisions with metropolitan

and micropolitan statistical areas. It would be appropriate to rank and compare

metropolitan divisions.

% N VALUE LABEL

2.3 18 20524

8.5 68 35004

44.3 354 35614

45.0 360

----- ---

100.0 800 cases

Data type: character

Record/columns: 1/65-69

**CENTRACT**: Census Tract - Primary (provided by MSG)

Census Tract is a small, relatively permanent sub-division of a county

(or county equivalent) used by the U.S. Bureau of the Census to collect

and tabulate Census data. A Census Tract generally contains between

1,500 and 8,000 people with an optimal size of 4,000 people. Census

Tracts do not cross County boundaries, but can cross city, township,

and town boundaries. Census Tract boundaries usually remain permanent

for about 10 years and change only at the onset of the decennial Census.

800 cases

Data type: character

Record/columns: 1/70-81

**nysregion**: NYS region (Upstate/Downstate)

NYS region (coded based on response to county or updown)

% % N VALUE LABEL

VALID ALL

50.0 50.0 400 1 Downstate

50.0 50.0 400 2 Upstate

----- ----- ---

100.0 100.0 800 cases

Min = 1 Mean = 1.500

Max = 2 Std Dev = .500

Median = 1.5 Variance = .250

(Based on 800 valid cases)

Data type: numeric

Minimum code defined as valid: 1

Missing-data codes: -1,-2

Record/columns: 1/125-126

**county**: NYS county (reported by R)

To make sure we're getting a mix of opinions from around the state,

would you please tell me which New York State county you currently live in?

NYC boroughs are: Brooklyn = Kings County

Manhattan = New York County

Staten Island = Richmond County

Bronx/Queens (county name is same as borough name)

800 cases (Range of valid codes: 1-121)

Min = 1 Mean = 58.703

Max = 121 Std Dev = 32.842

Median = 61 Variance = 1,078.612

(Based on 795 valid cases)

Data type: numeric

Minimum code defined as valid: 1

Missing-data codes: -1,-2

Record/columns: 1/127-129

**updown**: NYS region (reported by R)

It's very important that we make sure we're covering all regions of New York

State. So would you please at least confirm whether you live downstate

(that includes Brooklyn, Manhattan, Staten Island, Bronx, and Queens as well as

Long Island and Rockland and Westchester counties) or upstate (all other regions).

% % N VALUE LABEL

VALID ALL

100.0 0.4 3 1 Downstate

0.0 0.0 0 2 Upstate

0.0 0 -2 Do not know

0.4 3 -1 Refused

99.3 794 . (No Data)

----- ----- ---

100.0 100.0 800 cases

Min = 1 Mean = 1.000

Max = 1 Std Dev = .000

Median = 1 Variance = .000

(Based on 3 valid cases)

Data type: numeric

Minimum code defined as valid: 1

Missing-data codes: -1,-2

Record/columns: 1/130-131

**nysimp**: Most important NYS issue

In your opinion, what do you think is the single most important issue

facing New York State as a whole?

% % N VALUE LABEL

VALID ALL

4.5 4.4 35 1 Crime

7.6 7.5 60 2 Economic growth (economy)

7.3 7.1 57 3 Education

5.0 4.9 39 4 Employment

3.1 3.0 24 5 Environment

8.8 8.6 69 6 Health care

8.1 8.0 64 7 Housing (affordability/availability)

1.8 1.8 14 8 Immigration (foreign)

0.8 0.8 6 9 Land development

5.7 5.6 45 10 NYS government

1.4 1.4 11 11 NYS budget

0.6 0.6 5 12 People leaving NYS

0.5 0.5 4 13 Security/Threat of terrorism

20.4 20.0 160 14 Taxes (specify one specific type ...)

2.0 2.0 16 15 Climate change

2.7 2.6 21 16 Drug abuse

3.9 3.9 31 17 Transportation

1.0 1.0 8 18 Gun control (specify support or oppose)

3.4 3.4 27 19 Wealth inequality (including poverty)

11.5 11.3 90 20 Other (specify)

1.5 12 -2 Do not know

0.3 2 -1 Refused

----- ----- ---

100.0 100.0 800 cases

Min = 1 Mean = 10.322

Max = 20 Std Dev = 6.196

Median = 10 Variance = 38.387

(Based on 786 valid cases)

Data type: numeric

Minimum code defined as valid: 1

Missing-data codes: -1,-2

Record/columns: 1/132-133

**finpast**: Past year - Personal finances

We are interested in how people are getting along financially these

days. Would you say that you (and your household) are better off, worse off,

or just about the same financially as you were a year ago?

% % N VALUE LABEL

VALID ALL

18.5 18.5 148 1 Worse

60.1 60.1 481 2 About the same

21.4 21.4 171 3 Better

0.0 0 -2 Do not know

0.0 0 -1 Refused

----- ----- ---

100.0 100.0 800 cases

Min = 1 Mean = 2.029

Max = 3 Std Dev = .631

Median = 2 Variance = .398

(Based on 800 valid cases)

Data type: numeric

Minimum code defined as valid: 1

Missing-data codes: -1,-2

Record/columns: 1/134-135

**finfut**: Next year - Personal finances

Now looking ahead, do you think that a year from now you (and your

household) will be better off financially, worse off, or just about the same

as now?

% % N VALUE LABEL

VALID ALL

13.7 13.6 109 1 Worse

49.6 49.4 395 2 About the same

36.8 36.6 293 3 Better

0.4 3 -2 Do not know

0.0 0 -1 Refused

----- ----- ---

100.0 100.0 800 cases

Min = 1 Mean = 2.231

Max = 3 Std Dev = .672

Median = 2 Variance = .452

(Based on 797 valid cases)

Data type: numeric

Minimum code defined as valid: 1

Missing-data codes: -1,-2

Record/columns: 1/136-137

**econpast**: Past year - NYS economy

Thinking about the economy in New York State as a whole, would you say that

over the past year the state's economy has gotten better, stayed about the same,

or gotten worse?

% % N VALUE LABEL

VALID ALL

44.0 43.4 347 1 Worse

35.0 34.5 276 2 About the same

21.0 20.8 166 3 Better

1.3 10 -2 Do not know

0.1 1 -1 Refused

----- ----- ---

100.0 100.0 800 cases

Min = 1 Mean = 1.771

Max = 3 Std Dev = .774

Median = 2 Variance = .598

(Based on 789 valid cases)

Data type: numeric

Minimum code defined as valid: 1

Missing-data codes: -1,-2

Record/columns: 1/138-139

**econfut**: Next year - NYS economy

What about the next 12 months? Do you expect the economy in New York

State as a whole to get better, stay about the same, or get worse?

% % N VALUE LABEL

VALID ALL

37.5 37.0 296 1 Worse

38.8 38.3 306 2 About the same

23.7 23.4 187 3 Better

1.4 11 -2 Do not know

0.0 0 -1 Refused

----- ----- ---

100.0 100.0 800 cases

Min = 1 Mean = 1.862

Max = 3 Std Dev = .771

Median = 2 Variance = .594

(Based on 789 valid cases)

Data type: numeric

Minimum code defined as valid: 1

Missing-data codes: -1,-2

Record/columns: 1/140-141

**JAq1**: Fractions - Rate competence

How good are you at working with fractions?

% % N VALUE LABEL

VALID ALL

10.2 10.1 81 1 Not good at all

5.5 5.5 44 2

16.4 16.4 131 3

20.8 20.8 166 4

24.3 24.3 194 5

22.7 22.6 181 6 Extremely good

0.1 1 -2 Do not know

0.3 2 -1 Refused

----- ----- ---

100.0 100.0 800 cases

Min = 1 Mean = 4.118

Max = 6 Std Dev = 1.562

Median = 4 Variance = 2.441

(Based on 797 valid cases)

Data type: numeric

Minimum code defined as valid: 1

Missing-data codes: -1,-2

Record/columns: 1/166-167

**JAq2**: Percentages - Rate competence

How good are you at figuring out how much a shirt will cost if it is 25% off?

% % N VALUE LABEL

VALID ALL

4.2 4.1 33 1 Not good at all

2.0 2.0 16 2

6.2 6.1 49 3

10.9 10.9 87 4

20.9 20.8 166 5

55.8 55.5 444 6 Extremely good

0.3 2 -2 Do not know

0.4 3 -1 Refused

----- ----- ---

100.0 100.0 800 cases

Min = 1 Mean = 5.099

Max = 6 Std Dev = 1.324

Median = 6 Variance = 1.752

(Based on 795 valid cases)

Data type: numeric

Minimum code defined as valid: 1

Missing-data codes: -1,-2

Record/columns: 1/168-169

**JAq3**: Numerical information - Rate usefulness

How often do you find numerical information to be useful?

% % N VALUE LABEL

VALID ALL

3.4 3.4 27 1 Never

2.6 2.6 21 2

7.1 7.0 56 3

12.4 12.3 98 4

19.4 19.3 154 5

55.1 54.6 437 6 Very often

0.5 4 -2 Do not know

0.4 3 -1 Refused

----- ----- ---

100.0 100.0 800 cases

Min = 1 Mean = 5.071

Max = 6 Std Dev = 1.318

Median = 6 Variance = 1.737

(Based on 793 valid cases)

Data type: numeric

Minimum code defined as valid: 1

Missing-data codes: -1,-2

Record/columns: 1/170-171

**JAq4**: Rate health

In general, would you say your health is excellent, very good, good, fair,

or poor?

% % N VALUE LABEL

VALID ALL

13.4 13.4 107 1 Excellent

30.8 30.8 246 2 Very good

35.8 35.8 286 3 Good

16.4 16.4 131 4 Fair

3.8 3.8 30 5 Poor

0.0 0 -2 Do not know

0.0 0 -1 Refused

----- ----- ---

100.0 100.0 800 cases

Min = 1 Mean = 2.664

Max = 5 Std Dev = 1.022

Median = 3 Variance = 1.044

(Based on 800 valid cases)

Data type: numeric

Minimum code defined as valid: 1

Missing-data codes: -1,-2

Record/columns: 1/172-173

**SRIq1**: Societal pressure to attend college

Compared to 10 years ago, do you think the overall societal pressure to

attend college has increased, decreased, or remained the same?

(College includes 2-year degree programs and 4-year degree programs.

It does NOT include vocational or technical programs.)

% % N VALUE LABEL

VALID ALL

38.1 37.6 301 1 Increased greatly

19.6 19.4 155 2 Increased slightly

19.5 19.3 154 3 Remained the same

14.5 14.4 115 4 Decreased slightly

8.3 8.3 66 5 Decreased greatly

1.1 9 -2 Do not know

0.0 0 -1 Refused

----- ----- ---

100.0 100.0 800 cases

Min = 1 Mean = 2.355

Max = 5 Std Dev = 1.336

Median = 2 Variance = 1.784

(Based on 791 valid cases)

Data type: numeric

Minimum code defined as valid: 1

Missing-data codes: -1,-2

Record/columns: 1/174-175

**SRIq2**: Reason for change in societal pressure to attend college

What do you think is the primary reason for this change?

% % N VALUE LABEL

VALID ALL

100.0 78.9 631 1 Answer

0.6 5 -2 Do not know

0.1 1 -1 Refused

20.4 163 . (No Data)

----- ----- ---

100.0 100.0 800 cases

Min = 1 Mean = 1.000

Max = 1 Std Dev = .000

Median = 1 Variance = .000

(Based on 631 valid cases)

Data type: numeric

Minimum code defined as valid: 1

Missing-data codes: -1,-2

Record/columns: 1/176-177

**SRIq3**: Parents education

What is the highest level of education any one of your parents/guardians

has completed in school?

% % N VALUE LABEL

VALID ALL

8.2 8.1 65 1 None or grades 1-8

4.9 4.9 39 2 High school incomplete (grades 9-11)

35.9 35.5 284 3 High school graduate (grade 12 or GED certificate)

2.7 2.6 21 4 Technical, trade, or vocational school after high school

11.7 11.6 93 5 Some college, no 4-year degree (including 2 year Associate Degree)

18.1 17.9 143 6 College graduate (BS, BA, or other 4-year degree)

18.6 18.4 147 7 Post-graduate training or professional schooling after college

0.9 7 -2 Do not know

0.1 1 -1 Refused

----- ----- ---

100.0 100.0 800 cases

Min = 1 Mean = 4.332

Max = 7 Std Dev = 1.923

Median = 4 Variance = 3.699

(Based on 792 valid cases)

Data type: numeric

Minimum code defined as valid: 1

Missing-data codes: -1,-2

Record/columns: 1/178-179

**trst@corp**: Large corporations - Trustworthiness

Changing topics again, we have some questions about your level of trust

in various groups, particularly the government.

On a scale of 1 to 10, where 1 is untrustworthy and 10 is trustworthy, how

would you rate the trustworthiness of the following:

Large corporations

% % N VALUE LABEL

VALID ALL

20.3 20.1 161 1 Untrustworthy

6.6 6.5 52 2

12.5 12.4 99 3

8.6 8.5 68 4

23.8 23.6 189 5

9.7 9.6 77 6

8.7 8.6 69 7

5.7 5.6 45 8

1.3 1.3 10 9

2.9 2.9 23 10 Trustworthy

0.5 4 -2 Do not know

0.4 3 -1 Refused

----- ----- ---

100.0 100.0 800 cases

Min = 1 Mean = 4.293

Max = 10 Std Dev = 2.411

Median = 5 Variance = 5.813

(Based on 793 valid cases)

Data type: numeric

Minimum code defined as valid: 1

Missing-data codes: -1,-2

Record/columns: 1/297-298

**trst@bsns**: Local business - Trustworthiness

On a scale of 1 to 10, where 1 is untrustworthy and 10 is trustworthy, how

would you rate the trustworthiness of the following:

Local business

% % N VALUE LABEL

VALID ALL

2.5 2.5 20 1 Untrustworthy

1.4 1.4 11 2

3.8 3.8 30 3

2.8 2.8 22 4

18.1 18.0 144 5

10.2 10.1 81 6

19.6 19.5 156 7

21.3 21.1 169 8

9.8 9.8 78 9

10.6 10.5 84 10 Trustworthy

0.4 3 -2 Do not know

0.3 2 -1 Refused

----- ----- ---

100.0 100.0 800 cases

Min = 1 Mean = 6.808

Max = 10 Std Dev = 2.111

Median = 7 Variance = 4.455

(Based on 795 valid cases)

Data type: numeric

Minimum code defined as valid: 1

Missing-data codes: -1,-2

Record/columns: 1/299-300

**trst@lgov**: Local gov - Trustworthiness

On a scale of 1 to 10, where 1 is untrustworthy and 10 is trustworthy, how

would you rate the trustworthiness of the following:

Local government

% % N VALUE LABEL

VALID ALL

13.8 13.8 110 1 Untrustworthy

5.3 5.3 42 2

7.7 7.6 61 3

9.2 9.1 73 4

23.7 23.6 189 5

11.3 11.3 90 6

12.8 12.8 102 7

10.2 10.1 81 8

2.8 2.8 22 9

3.4 3.4 27 10 Trustworthy

0.0 0 -2 Do not know

0.4 3 -1 Refused

----- ----- ---

100.0 100.0 800 cases

Min = 1 Mean = 4.999

Max = 10 Std Dev = 2.432

Median = 5 Variance = 5.916

(Based on 797 valid cases)

Data type: numeric

Minimum code defined as valid: 1

Missing-data codes: -1,-2

Record/columns: 1/301-302

**trst@sgov**: State gov - Trustworthiness

On a scale of 1 to 10, where 1 is untrustworthy and 10 is trustworthy, how

would you rate the trustworthiness of the following:

State government

% % N VALUE LABEL

VALID ALL

20.4 20.3 162 1 Untrustworthy

8.0 8.0 64 2

10.6 10.5 84 3

10.8 10.8 86 4

22.1 22.0 176 5

10.1 10.0 80 6

8.7 8.6 69 7

4.8 4.8 38 8

1.9 1.9 15 9

2.8 2.8 22 10 Trustworthy

0.1 1 -2 Do not know

0.4 3 -1 Refused

----- ----- ---

100.0 100.0 800 cases

Min = 1 Mean = 4.256

Max = 10 Std Dev = 2.415

Median = 5 Variance = 5.834

(Based on 796 valid cases)

Data type: numeric

Minimum code defined as valid: 1

Missing-data codes: -1,-2

Record/columns: 1/303-304

**trst@union**: Labor unions - Trustworthiness

On a scale of 1 to 10, where 1 is untrustworthy and 10 is trustworthy, how

would you rate the trustworthiness of the following:

Labor unions

% % N VALUE LABEL

VALID ALL

8.9 8.8 70 1 Untrustworthy

5.5 5.4 43 2

8.6 8.5 68 3

9.4 9.3 74 4

21.9 21.5 172 5

11.2 11.0 88 6

13.9 13.6 109 7

11.7 11.5 92 8

3.6 3.5 28 9

5.5 5.4 43 10 Trustworthy

1.1 9 -2 Do not know

0.5 4 -1 Refused

----- ----- ---

100.0 100.0 800 cases

Min = 1 Mean = 5.368

Max = 10 Std Dev = 2.425

Median = 5 Variance = 5.879

(Based on 787 valid cases)

Data type: numeric

Minimum code defined as valid: 1

Missing-data codes: -1,-2

Record/columns: 1/305-306

**trst@media**: News media - Trustworthiness

On a scale of 1 to 10, where 1 is untrustworthy and 10 is trustworthy, how

would you rate the trustworthiness of the following:

News media

% % N VALUE LABEL

VALID ALL

21.7 21.6 173 1 Untrustworthy

8.1 8.1 65 2

8.8 8.8 70 3

7.9 7.9 63 4

19.6 19.6 157 5

9.0 9.0 72 6

8.8 8.8 70 7

8.3 8.3 66 8

3.3 3.3 26 9

4.6 4.6 37 10 Trustworthy

0.0 0 -2 Do not know

0.1 1 -1 Refused

----- ----- ---

100.0 100.0 800 cases

Min = 1 Mean = 4.511

Max = 10 Std Dev = 2.694

Median = 5 Variance = 7.258

(Based on 799 valid cases)

Data type: numeric

Minimum code defined as valid: 1

Missing-data codes: -1,-2

Record/columns: 1/307-308

**trst@ppl**: Other people - Trustworthiness

On a scale of 1 to 10, where 1 is untrustworthy and 10 is trustworthy, how

would you rate the trustworthiness of the following:

Other people in general

% % N VALUE LABEL

VALID ALL

4.9 4.9 39 1 Untrustworthy

3.2 3.1 25 2

6.1 6.0 48 3

4.4 4.4 35 4

26.6 26.3 210 5

11.9 11.8 94 6

18.6 18.4 147 7

16.2 16.0 128 8

4.8 4.8 38 9

3.3 3.3 26 10 Trustworthy

0.9 7 -2 Do not know

0.4 3 -1 Refused

----- ----- ---

100.0 100.0 800 cases

Min = 1 Mean = 5.876

Max = 10 Std Dev = 2.125

Median = 6 Variance = 4.514

(Based on 790 valid cases)

Data type: numeric

Minimum code defined as valid: 1

Missing-data codes: -1,-2

Record/columns: 1/309-310

**trst@intrnt**: Internet - Trustworthiness

On a scale of 1 to 10, where 1 is untrustworthy and 10 is trustworthy, how

would you rate the trustworthiness of the following:

The Internet

% % N VALUE LABEL

VALID ALL

14.4 14.3 114 1 Untrustworthy

8.9 8.8 70 2

11.5 11.4 91 3

10.4 10.3 82 4

27.6 27.3 218 5

8.9 8.8 70 6

8.5 8.4 67 7

5.8 5.8 46 8

1.0 1.0 8 9

2.9 2.9 23 10 Trustworthy

1.0 8 -2 Do not know

0.4 3 -1 Refused

----- ----- ---

100.0 100.0 800 cases

Min = 1 Mean = 4.441

Max = 10 Std Dev = 2.283

Median = 5 Variance = 5.214

(Based on 789 valid cases)

Data type: numeric

Minimum code defined as valid: 1

Missing-data codes: -1,-2

Record/columns: 1/311-312

**employ**: Employment status

Some of our remaining questions depend on your employment status.

Last week, did you do any work for either pay or profit? Include any job

from which you were on vacation, temporarily absent, or on layoff.

% % N VALUE LABEL

VALID ALL

59.3 59.1 473 1 Yes

19.7 19.6 157 2 No

16.8 16.8 134 3 Retired

3.3 3.3 26 4 Disabled

1.0 1.0 8 5 Unable to work

0.3 2 -1 Refused

----- ----- ---

100.0 100.0 800 cases

Min = 1 Mean = 1.670

Max = 5 Std Dev = .935

Median = 1 Variance = .874

(Based on 798 valid cases)

Data type: numeric

Minimum code defined as valid: 1

Missing-data codes: -1,-2

Record/columns: 1/319-320

**jbtype**: Main job type

Which of the following best describes your main job? By main job we

mean the one at which you usually work the most hours.

% % N VALUE LABEL

VALID ALL

73.7 43.5 348 1 Full-time, all year round

19.3 11.4 91 2 Part-time, all year round

1.5 0.9 7 3 Temporary

3.0 1.8 14 4 Seasonal or part year

2.5 1.5 12 5 Contract or on call

0.0 0 -2 Do not know

0.1 1 -1 Refused

40.9 327 . (No Data)

----- ----- ---

100.0 100.0 800 cases

Min = 1 Mean = 1.413

Max = 5 Std Dev = .870

Median = 1 Variance = .757

(Based on 472 valid cases)

Data type: numeric

Minimum code defined as valid: 1

Missing-data codes: -1,-2

Record/columns: 1/321-322

**hrswrk**: Hours worked last week

How many hours did you work last week, at all jobs?

800 cases (Range of valid codes: 0-100)

Min = 0 Mean = 40.212

Max = 100 Std Dev = 15.389

Median = 40 Variance = 236.830

(Based on 472 valid cases)

Data type: numeric

Minimum code defined as valid: 0

Missing-data codes: -1,-2

Record/columns: 1/323-325

**slfempl**: Self-employed

Are you self-employed without employees (i.e. consultant, freelancer)

on your main job?

% % N VALUE LABEL

VALID ALL

83.3 49.3 394 0 No

16.7 9.9 79 1 Yes

0.0 0 -1 Refused

40.9 327 . (No Data)

----- ----- ---

100.0 100.0 800 cases

Min = 0 Mean = .167

Max = 1 Std Dev = .373

Median = 0 Variance = .139

(Based on 473 valid cases)

Data type: numeric

Minimum code defined as valid: 0

Missing-data codes: -1,-2

Record/columns: 1/326-327

**lkwork**: Looking for new work

In the last four weeks have you looked for new work or a new job?

% % N VALUE LABEL

VALID ALL

78.9 78.0 624 0 No

21.1 20.9 167 1 Yes

0.1 1 -1 Refused

1.0 8 . (No Data)

----- ----- ---

100.0 100.0 800 cases

Min = 0 Mean = .211

Max = 1 Std Dev = .408

Median = 0 Variance = .167

(Based on 791 valid cases)

Data type: numeric

Minimum code defined as valid: 0

Missing-data codes: -1,-2

Record/columns: 1/328-329

**bornus**: Born in US or to an American

We're almost done! I have just a few demographic questions, to make sure

we're getting opinions from people with a variety of backgrounds.

Were you born in the United States or in one of its territories (Puerto Rico,

Guam, the Virgin Islands), or abroad to an American parent?

% % N VALUE LABEL

VALID ALL

81.5 81.4 651 1 Born in the United States (or a territory)

1.3 1.3 10 2 Born abroad to at least one American parent

17.3 17.3 138 3 Not born in the United States (nor a territory) nor to an American parent

0.1 1 -1 Refused

----- ----- ---

100.0 100.0 800 cases

Min = 1 Mean = 1.358

Max = 3 Std Dev = .759

Median = 1 Variance = .576

(Based on 799 valid cases)

Data type: numeric

Minimum code defined as valid: 1

Missing-data codes: -1,-2

Record/columns: 1/330-331

**yob**: Year of birth

What year were you born?

800 cases (Range of valid codes: 1,921-2,001)

Min = 1,921 Mean = 1,970.516

Max = 2,001 Std Dev = 17.972

Median = 1,969.5 Variance = 322.979

(Based on 794 valid cases)

Data type: numeric

Minimum code defined as valid: 1910

Missing-data codes: -1,-2

Record/columns: 1/332-335

**age**: Age (computed from yob)

800 cases (Range of valid codes: 18-98)

Min = 18 Mean = 48.484

Max = 98 Std Dev = 17.972

Median = 49.5 Variance = 322.979

(Based on 794 valid cases)

Data type: numeric

Missing-data codes: -1,-2

Record/columns: 1/336-338

**Zipcode**: ZIP code

What is your ZIP code?

800 cases (Range of valid codes: 32-91267)

Min = 32 Mean = 12,525.781

Max = 91,267 Std Dev = 4,370.823

Median = 11,851 Variance = 19,104,096.718

(Based on 784 valid cases)

Data type: numeric

Minimum code defined as valid: 0

Missing-data code: 99999

Record/columns: 1/339-343

**married**: Marital status

Are you married, divorced, separated, widowed, or single?

% % N VALUE LABEL

VALID ALL

48.2 48.0 384 1 Married

9.5 9.5 76 2 Divorced

2.0 2.0 16 3 Separated

4.0 4.0 32 4 Widowed

36.3 36.1 289 5 Single

0.0 0.0 0 6 Other (specify ...)

0.4 3 -1 Refused

----- ----- ---

100.0 100.0 800 cases

Min = 1 Mean = 2.706

Max = 5 Std Dev = 1.852

Median = 2 Variance = 3.431

(Based on 797 valid cases)

Data type: numeric

Minimum code defined as valid: 1

Missing-data codes: -1,-2

Record/columns: 1/344-345

**ideo**: Social ideology

When it comes to social issues, do you usually think of yourself as extremely

liberal, liberal, slightly liberal, moderate or middle of the road, slightly

conservative, conservative, or extremely conservative?

% % N VALUE LABEL

VALID ALL

7.2 7.0 56 1 Extremely liberal

16.8 16.4 131 2 Liberal

9.7 9.5 76 3 Slightly liberal

36.4 35.5 284 4 Moderate or middle of the road

10.0 9.8 78 5 Slightly conservative

13.7 13.4 107 6 Conservative

6.3 6.1 49 7 Extremely conservative

1.9 15 -2 Do not know

0.5 4 -1 Refused

----- ----- ---

100.0 100.0 800 cases

Min = 1 Mean = 3.914

Max = 7 Std Dev = 1.619

Median = 4 Variance = 2.622

(Based on 781 valid cases)

Data type: numeric

Minimum code defined as valid: 1

Missing-data codes: -1,-2

Record/columns: 1/346-347

**party**: Political party

Generally speaking, when it comes to political parties in the United States,

how would you best describe yourself?

% % N VALUE LABEL

VALID ALL

23.3 22.9 183 1 Strong Democrat

13.0 12.8 102 2 Not very strong Democrat

12.8 12.6 101 3 Independent, close to Democrat

20.7 20.4 163 4 Independent (close to Neither)

7.0 6.9 55 5 Independent, close to Republican

8.4 8.3 66 6 Not very strong Republican

11.3 11.1 89 7 Strong Republican

3.4 3.4 27 8 Other party affiliation (specify ...)

0.8 6 -2 Do not know

1.0 8 -1 Refused

----- ----- ---

100.0 100.0 800 cases

Min = 1 Mean = 3.628

Max = 8 Std Dev = 2.141

Median = 4 Variance = 4.585

(Based on 786 valid cases)

Data type: numeric

Minimum code defined as valid: 1

Missing-data codes: -1,-2

Record/columns: 1/348-349

**educ**: Education level

What is the last grade or class that you completed in school?

% % N VALUE LABEL

VALID ALL

2.4 2.4 19 1 None, or grades 1-8

3.6 3.6 29 2 High school incomplete (grades 9-11)

17.0 17.0 136 3 High school graduate (grade 12 or GED certificate)

2.3 2.3 18 4 Technical, trade, or vocational school after high school

25.8 25.8 206 5 Some college, no 4-year degree (including 2-year Associate degree)

28.6 28.5 228 6 College graduate (BS, BA, or other 4-year degree)

20.3 20.3 162 7 Post-graduate training or professional schooling after college

0.3 2 -1 Refused

----- ----- ---

100.0 100.0 800 cases

Min = 1 Mean = 5.124

Max = 7 Std Dev = 1.580

Median = 5 Variance = 2.498

(Based on 798 valid cases)

Data type: numeric

Minimum code defined as valid: 1

Missing-data codes: -1,-2

Record/columns: 1/350-351

**SRIq4**: Reason NOT to attend college

What is the primary reason you decided NOT to pursue a college degree?

% % N VALUE LABEL

VALID ALL

4.1 1.0 8 1 Lack of job prospects

25.6 6.3 50 2 Affordability

8.7 2.1 17 3 Time investment

61.5 15.0 120 4 Some other reason (specify...)

0.6 5 -2 Do not know

0.3 2 -1 Refused

74.8 598 . (No Data)

----- ----- ---

100.0 100.0 800 cases

Min = 1 Mean = 3.277

Max = 4 Std Dev = .982

Median = 4 Variance = .964

(Based on 195 valid cases)

Data type: numeric

Minimum code defined as valid: 1

Missing-data codes: -1,-2

Record/columns: 1/352-353

**SRIq5**: Reason to attend college

What is the primary reason you decided to pursue a college degree?

% % N VALUE LABEL

VALID ALL

45.4 33.6 269 1 Promise of job prospects

14.0 10.4 83 2 Familial or societal pressures

27.2 20.1 161 3 Pursuit of knowledge

13.3 9.9 79 4 Some other reason (specify...)

0.1 1 -2 Do not know

0.4 3 -1 Refused

25.5 204 . (No Data)

----- ----- ---

100.0 100.0 800 cases

Min = 1 Mean = 2.084

Max = 4 Std Dev = 1.120

Median = 2 Variance = 1.255

(Based on 592 valid cases)

Data type: numeric

Minimum code defined as valid: 1

Missing-data codes: -1,-2

Record/columns: 1/354-355

**hisp**: Hispanic or Latino

Are you, yourself, of Hispanic origin or descent, such as Mexican, Puerto

Rican, Cuban, or some other Spanish background?

% % N VALUE LABEL

VALID ALL

84.2 83.9 671 0 No

15.8 15.8 126 1 Yes

0.4 3 -1 Refused

----- ----- ---

100.0 100.0 800 cases

Min = 0 Mean = .158

Max = 1 Std Dev = .365

Median = 0 Variance = .133

(Based on 797 valid cases)

Data type: numeric

Minimum code defined as valid: 0

Missing-data codes: -1,-2

Record/columns: 1/356-357

**race@white**: White/Caucasian - Race

What best describes your race? Please tell me yes or no for each of

the following races:

White or Caucasian

% % N VALUE LABEL

VALID ALL

27.5 27.3 218 0 No

72.5 72.0 576 1 Yes

0.8 6 -1 Refused

----- ----- ---

100.0 100.0 800 cases

Min = 0 Mean = .725

Max = 1 Std Dev = .447

Median = 1 Variance = .199

(Based on 794 valid cases)

Data type: numeric

Minimum code defined as valid: 0

Missing-data codes: -1,-2

Record/columns: 1/358-359

**race@black**: Black/African American - Race

What best describes your race? Please tell me yes or no for each of

the following races:

Black or African-American

% % N VALUE LABEL

VALID ALL

84.4 83.8 670 0 No

15.6 15.5 124 1 Yes

0.8 6 -1 Refused

----- ----- ---

100.0 100.0 800 cases

Min = 0 Mean = .156

Max = 1 Std Dev = .363

Median = 0 Variance = .132

(Based on 794 valid cases)

Data type: numeric

Minimum code defined as valid: 0

Missing-data codes: -1,-2

Record/columns: 1/360-361

**race@natam**: American Indian/Aleut/Eskimo - Race

What best describes your race? Please tell me yes or no for each of

the following races:

American Indian, Aleut, Eskimo

% % N VALUE LABEL

VALID ALL

95.8 95.1 761 0 No

4.2 4.1 33 1 Yes

0.8 6 -1 Refused

----- ----- ---

100.0 100.0 800 cases

Min = 0 Mean = .042

Max = 1 Std Dev = .200

Median = 0 Variance = .040

(Based on 794 valid cases)

Data type: numeric

Minimum code defined as valid: 0

Missing-data codes: -1,-2

Record/columns: 1/362-363

**race@asian**: Asian/Pacific Islander - Race

What best describes your race? Please tell me yes or no for each of

the following races:

Asian or Pacific Islander

% % N VALUE LABEL

VALID ALL

92.9 92.3 738 0 No

7.1 7.0 56 1 Yes

0.8 6 -1 Refused

----- ----- ---

100.0 100.0 800 cases

Min = 0 Mean = .071

Max = 1 Std Dev = .256

Median = 0 Variance = .066

(Based on 794 valid cases)

Data type: numeric

Minimum code defined as valid: 0

Missing-data codes: -1,-2

Record/columns: 1/364-365

**race@other**: Other - Race

What best describes your race? Please tell me yes or no for each of

the following races:

Other (specify...)

% % N VALUE LABEL

VALID ALL

100.0 6.4 51 1 Yes (specify ...)

1.4 11 -1 Refused

92.3 738 . (No Data)

----- ----- ---

100.0 100.0 800 cases

Min = 1 Mean = 1.000

Max = 1 Std Dev = .000

Median = 1 Variance = .000

(Based on 51 valid cases)

Data type: numeric

Minimum code defined as valid: 1

Missing-data codes: -1,-2

Record/columns: 1/366-367

**relig**: Religious affiliation

What is your religious preference? Is it Protestant, Catholic,

Christian Orthodox, Jewish, Muslim, some other religion, or no religion?

% % N VALUE LABEL

VALID ALL

27.3 27.0 216 1 Protestant

28.4 28.1 225 2 Catholic

4.5 4.5 36 3 Christian Orthodox

5.2 5.1 41 4 Jewish

2.7 2.6 21 5 Muslim

4.3 4.3 34 6 Other non-Christian religion (specify ...)

27.7 27.4 219 7 No religion / Atheist / Agnostic

1.0 8 -1 Refused

----- ----- ---

100.0 100.0 800 cases

Min = 1 Mean = 3.510

Max = 7 Std Dev = 2.468

Median = 2 Variance = 6.091

(Based on 792 valid cases)

Data type: numeric

Minimum code defined as valid: 1

Missing-data codes: -1,-2

Record/columns: 1/368-369

**church**: How often attend religious services

Aside from weddings and funerals, how often do you attend religious services?

More than once a week, once a week, once or twice a month, a few times a year,

seldom, or never?

% % N VALUE LABEL

VALID ALL

7.4 7.4 59 1 More than once a week

18.8 18.8 150 2 Once a week

11.7 11.6 93 3 Once or twice a month

17.6 17.5 140 4 A few times a year

17.0 16.9 135 5 Seldom

27.5 27.4 219 6 Never

0.5 4 -1 Refused

----- ----- ---

100.0 100.0 800 cases

Min = 1 Mean = 4.004

Max = 6 Std Dev = 1.677

Median = 4 Variance = 2.811

(Based on 796 valid cases)

Data type: numeric

Minimum code defined as valid: 1

Missing-data codes: -1,-2

Record/columns: 1/370-371

**hhsize@65**: # Adults 65+ in household

How many total people, including yourself, in your household are ...

Adults (65 and older)

% % N VALUE LABEL

VALID ALL

65.7 65.5 524 0

17.9 17.9 143 1

14.4 14.4 115 2

1.3 1.3 10 3

0.3 0.3 2 4

0.5 0.5 4 5

0.3 2 -1 Refused

----- ----- ---

100.0 100.0 800 cases

Min = 0 Mean = .540

Max = 5 Std Dev = .862

Median = 0 Variance = .743

(Based on 798 valid cases)

Data type: numeric

Minimum code defined as valid: 0

Missing-data codes: -1,-2

Record/columns: 1/372-373

**hhsize@1864**: # Adults 18-64 in household

How many total people, including yourself, in your household are ...

Adults (18-64)

% % N VALUE LABEL

VALID ALL

17.1 17.0 136 0

22.7 22.6 181 1

35.0 34.9 279 2

13.9 13.9 111 3

7.3 7.3 58 4

3.0 3.0 24 5

0.3 0.3 2 6

0.1 0.1 1 7

0.5 0.5 4 8

0.1 0.1 1 10

0.4 3 -1 Refused

----- ----- ---

100.0 100.0 800 cases

Min = 0 Mean = 1.863

Max = 10 Std Dev = 1.388

Median = 2 Variance = 1.927

(Based on 797 valid cases)

Data type: numeric

Minimum code defined as valid: 0

Missing-data codes: -1,-2

Record/columns: 1/374-375

**hhsize@chld**: # Children in household

How many total people, including yourself, in your household are ...

Children (under 18)

% % N VALUE LABEL

VALID ALL

66.2 66.0 528 0

13.8 13.8 110 1

11.8 11.8 94 2

5.5 5.5 44 3

2.0 2.0 16 4

0.4 0.4 3 5

0.1 0.1 1 6

0.1 0.1 1 7

0.4 3 -1 Refused

----- ----- ---

100.0 100.0 800 cases

Min = 0 Mean = .655

Max = 7 Std Dev = 1.096

Median = 0 Variance = 1.201

(Based on 797 valid cases)

Data type: numeric

Minimum code defined as valid: 0

Missing-data codes: -1,-2

Record/columns: 1/376-377

**hhince**: Exact household income

For statistical purposes, last year (that is in 2018) what was your

total household income from all sources, before taxes?

800 cases (Range of valid codes: 8,000-850,000)

Min = 8,000 Mean = 102,413.246

Max = 850,000 Std Dev = 96,713.381

Median = 80,000 Variance = 9,353,477,973.741

(Based on 536 valid cases)

Data type: numeric

Minimum code defined as valid: 8000

Missing-data codes: -1,-2

Record/columns: 1/378-384

**hhinc50k**: Over/Under $50k - Household income

Instead of a specific number, please tell me if your total household income

in 2018 was under or over $50,000.

% % N VALUE LABEL

VALID ALL

43.6 12.8 102 5 Under $50,000

56.4 16.5 132 6 $50,000 or over

1.0 8 -2 Do not know

2.8 22 -1 Refused

67.0 536 . (No Data)

----- ----- ---

100.0 100.0 800 cases

Min = 5 Mean = 5.564

Max = 6 Std Dev = .497

Median = 6 Variance = .247

(Based on 234 valid cases)

Data type: numeric

Minimum code defined as valid: 5

Missing-data codes: -1,-2

Record/columns: 1/386-387

**hhincu**: Range under $50k - Household income

Instead of a specific number, please tell me if your total household income

in 2018 was under or over $50,000.

And was it:

% % N VALUE LABEL

VALID ALL

27.8 2.5 20 1 Less than $10,000

22.2 2.0 16 2 10 to under $20,000

18.1 1.6 13 3 20 to under $30,000

16.7 1.5 12 4 30 to under $40,000

15.3 1.4 11 5 40 to under $50,000

2.0 16 -2 Do not know

1.8 14 -1 Refused

87.3 698 . (No Data)

----- ----- ---

100.0 100.0 800 cases

Min = 1 Mean = 2.694

Max = 5 Std Dev = 1.430

Median = 2.5 Variance = 2.046

(Based on 72 valid cases)

Data type: numeric

Minimum code defined as valid: 1

Missing-data codes: -1,-2

Record/columns: 1/388-389

**hhinco**: Range over $50k - Household income

Instead of a specific number, please tell me if your total household income

in 2018 was under or over $50,000.

And was it:

% % N VALUE LABEL

VALID ALL

34.0 4.3 34 6 50 to under $75,000

20.0 2.5 20 7 75 to under $100,000

23.0 2.9 23 8 100 to under $150,000

23.0 2.9 23 9 $150,000 or more

1.1 9 -2 Do not know

2.9 23 -1 Refused

83.5 668 . (No Data)

----- ----- ---

100.0 100.0 800 cases

Min = 6 Mean = 7.350

Max = 9 Std Dev = 1.175

Median = 7 Variance = 1.381

(Based on 100 valid cases)

Data type: numeric

Minimum code defined as valid: 6

Missing-data codes: -1,-2

Record/columns: 1/390-391

**hhinc**: Household income - Final coded value

The coded value for household income is a single scale with the best response

obtained from all of the household income items (hhince, hhinc50k, hhincu, hhinco

If available, the exact household income (from hhince) is coded according to the

scale below.

Otherwise, if an income range is available (from hhincu or hhinco), it is

copied to this variable.

Otherwise, if only a response to hhinc50k is available, incomes of "Under $50,000"

are coded as 5 ($40,000 to under $50,000) and incomes of "$50,000 or over" are coded

as 6 ($50,000 to under $75,000).

% % N VALUE LABEL

VALID ALL

3.1 3.0 24 1 Less than $10,000

5.5 5.3 42 2 10,000 to under $20,000

7.0 6.8 54 3 20,000 to under $30,000

7.8 7.5 60 4 30,000 to under $40,000

9.5 9.1 73 5 40,000 to under $50,000

20.1 19.4 155 6 50,000 to under $75,000

13.9 13.4 107 7 75,000 to under $100,000

14.5 14.0 112 8 100,000 to under $150,000

18.6 17.9 143 9 More than $150,000

3.8 30 . (No Data)

----- ----- ---

100.0 100.0 800 cases

Min = 1 Mean = 6.152

Max = 9 Std Dev = 2.251

Median = 6 Variance = 5.065

(Based on 770 valid cases)

Data type: numeric

Missing-data codes: -1,-2

Record/columns: 1/392-393

**gender**: Gender

And finally, what is your gender?

% % N VALUE LABEL

VALID ALL

50.5 50.4 403 1 Male

49.2 49.1 393 2 Female

0.3 0.3 2 3 Other (specify...)

0.3 2 -1 Refused

----- ----- ---

100.0 100.0 800 cases

Min = 1 Mean = 1.497

Max = 3 Std Dev = .505

Median = 1 Variance = .255

(Based on 798 valid cases)

Data type: numeric

Minimum code defined as valid: 1

Missing-data codes: -1,-2

Record/columns: 1/394-395

**datecomp@month**: Month - Date of completion

Survey date completion

Month

% % N VALUE LABEL

VALID ALL

18.3 18.3 146 2

56.1 56.1 449 3

25.6 25.6 205 4

----- ----- ---

100.0 100.0 800 cases

Min = 2 Mean = 3.074

Max = 4 Std Dev = .659

Median = 3 Variance = .434

(Based on 800 valid cases)

Data type: numeric

Missing-data codes: -1,-2

Record/columns: 1/396-397

**datecomp@day**: Day - Date of completion

Survey date completion

Day

% % N VALUE LABEL

VALID ALL

2.1 2.1 17 1

2.4 2.4 19 2

3.1 3.1 25 3

3.8 3.8 30 4

2.5 2.5 20 5

3.0 3.0 24 6

4.4 4.4 35 7

1.8 1.8 14 8

3.1 3.1 25 9

5.5 5.5 44 10

6.1 6.1 49 11

2.5 2.5 20 12

2.8 2.8 22 13

2.3 2.3 18 14

1.8 1.8 14 15

2.6 2.6 21 16

4.6 4.6 37 17

3.9 3.9 31 18

4.4 4.4 35 19

4.6 4.6 37 20

3.6 3.6 29 21

1.3 1.3 10 22

3.0 3.0 24 23

5.5 5.5 44 24

4.1 4.1 33 25

3.9 3.9 31 26

4.8 4.8 38 27

3.8 3.8 30 28

1.3 1.3 10 30

1.8 1.8 14 31

----- ----- ---

100.0 100.0 800 cases

Min = 1 Mean = 15.799

Max = 31 Std Dev = 8.312

Median = 17 Variance = 69.082

(Based on 800 valid cases)

Data type: numeric

Missing-data codes: -1,-2

Record/columns: 1/398-399

**datecomp@year**: Year - Date of completion

Survey date completion

Year

% % N VALUE LABEL

VALID ALL

100.0 100.0 800 2019

----- ----- ---

100.0 100.0 800 cases

Min = 2,019 Mean = 2,019.000

Max = 2,019 Std Dev = .000

Median = 2,019 Variance = .000

(Based on 800 valid cases)

Data type: numeric

Missing-data codes: -1,-2

Record/columns: 1/400-403

**datecomp@time**: Time started

Time started

800 cases (Range of valid codes: 814-2102)

Min = 814 Mean = 1,851.549

Max = 2,102 Std Dev = 207.604

Median = 1,907 Variance = 43,099.510

(Based on 800 valid cases)

Data type: numeric

Missing-data codes: -1,-2

Record/columns: 1/404-407

**survlang**: Survey language

Interviewer: In which language was this survey conducted?

% % N VALUE LABEL

VALID ALL

100.0 96.5 772 1 English

3.5 28 8 Spanish

----- ----- ---

100.0 100.0 800 cases

Min = 1 Mean = 1.000

Max = 1 Std Dev = .000

Median = 1 Variance = .000

(Based on 772 valid cases)

Data type: numeric

Minimum code defined as valid: 1

Missing-data code: 8

Record/column: 1/408
